# Schistosomiasis and HIV-1 viral load in HIV-infected outpatients with immunological failure in Tanzania: a case-control study

**DOI:** 10.1186/s12879-019-3876-8

**Published:** 2019-03-12

**Authors:** Peter Masikini, Soledad Colombe, Amon Marti, Bernard Desderius, Claudia J. de Dood, Paul L. A. M. Corstjens, Govert J. van Dam, Mwanaisha Seugendo, Samuel Kalluvya, Jennifer A. Downs

**Affiliations:** 1 Department of Medicine, Bugando Medical Centre, Mwanza, Tanzania; 2 Center for Global Health, Department of Medicine, Weill Cornell Medicine, New York, USA; 3 Department of Cell and Chemical Biology, Leiden University Medical Center, Leiden, Netherlands; 4 Department of Parasitology, Leiden University Medical Center, Leiden, Netherlands

**Keywords:** *Schistosoma* sp., Circulating anodic antigen, HIV-1, Viral load, Tanzania, ART, Treatment failure

## Abstract

**Background:**

*Schistosoma* sp. infection has been shown to interact with HIV-1 by modifying susceptibility to the virus and impacting AIDS outcome, but very little is known about the potential impact of *Schistosoma* sp. infection on the efficiency of antiretroviral treatment (ART) in HIV-1 infected individuals. One study suggested increased immunological failure in patients infected with schistosomes compared to those uninfected. To our knowledge, no report exists on the virological response to ART in schistosome-infected individuals. In addition, viral load in HIV-1 infected individuals changes over the course of the HIV infection. This study assessed the impact of HIV-1/*Schistosoma* sp. co-infections on viral load in people with immunological failure on ART, taking into account the duration of HIV-1 infection.

**Methods:**

We enrolled HIV-1 infected Tanzanian adults over 18 years of age who had used first line ART for more than 6 months and were identified to have immunological failure by the WHO criteria (50% drop from peak CD4 count, or CD4 count equal to or below baseline after 6 months of ART, or CD4 count below 100cells/mm^3^ after 1 year of ART). Patients were also tested for schistosome infection by microscopy for ova in urine and stool and by circulating anodic antigen (CAA) levels in serum. The duration of HIV-1 infection was calculated using baseline CD4+ T-cell (CD4) counts determined at enrollment. Univariable and multivariable analyses were conducted to compare viral loads in schistosome infected and uninfected patients.

**Results:**

A total of 188 patients were enrolled. After univariable analysis, female sex, lower peak CD4 counts, lower current CD4 counts, anemia, and shorter time infected with HIV-1 were all significantly associated with higher viral load. Schistosome infection was not associated with viral load even after adjusting for sex, current CD4 counts and duration of HIV-1 infection.

**Conclusions:**

The current study of HIV-infected patients with immunological failure on ART suggests that once ART is introduced, ART is the dominant driver of viral load and schistosome infection may no longer have an impact.

## Background

*Schistosoma* sp. and HIV are co-endemic globally and an estimated 6 million individuals are co-infected worldwide [1, 2]. *Schistosoma* sp. infection has been shown to increase susceptibility to HIV and to impact HIV/AIDS outcomes [3–8]. It has been hypothesized that schistosome-induced down-regulation of T helper (Th)-1 immune responses may permit increased viral replication, and that T regulatory cells may also play an important role in the observed interactions.

Availability of antiretroviral therapy (ART) is increasing, with 61% of adults infected with HIV being on antiretroviral treatment in sub-Saharan Africa [9]. Yet very little is known about the impact of *Schistosoma* sp. infection on the effectiveness of ART. One study from Tanzania showed schistosome infection to be significantly associated with immunological failure and poorer CD4+ T-cells (CD4) count gain with ART [10]. However that study did not investigate whether the immunological failure was associated with concomitant increase of viral load (virological failure). Results from human studies of schistosome infection and HIV-1 RNA viral loads are variable, with some showing lower and some showing higher viral replication during schistosome co-infection [3, 11–18].

Our group has recently demonstrated that people with schistosome infections have higher HIV-1 viral load set-points shortly following HIV acquisition, yet that in chronic HIV-schistosome co-infection, viral loads are lower than in those with HIV infection alone [19]. To our knowledge, no report exists on the virological response to ART in schistosome-infected individuals. Furthermore, controlling for duration of HIV-1 infection appears to be a critical factor to consider in studies of HIV-1 and schistosome co-infection, since HIV-1 viral load differs over the course of infection [19, 20].

In the current study, we aimed to assess the impact of HIV-1/*Schistosoma* sp. co-infections on viral load in HIV-1 infected people with immunological failure on ART, taking into account the duration of HIV-1 infection.

## Methods

### Study participants and enrollment

This study was conducted in HIV outpatient clinics at Bugando Medical Centre (BMC) and Sekou Toure Hospital in Mwanza. The participants were HIV-1 infected adults over 18 years of age, who had used first line ART for more than 6 months and were identified to have immunological failure by the World Health Organization (WHO) criteria. Patients on stavudine-containing regimens were excluded due to this drug’s known association with treatment failure [21, 22]. Eligible patients provided urine and stool samples for schistosomiasis testing by microscopy as well as serum for quantitation of schistosome circulating anodic antigen (CAA). Plasma was also collected for viral load measurement. Additional information was extracted from the HIV clinic database and the patient’s chart.

### Laboratory methods

Tests for schistosome infections were performed at the National Institute of Medical Research in Mwanza, Tanzania. Microscopic examinations were performed on 10 mL of urine (for *S. haematobium*) by the filtration technique and on stool (for *S. mansoni*) following the Kato Katz method. CAA testing was performed as previously described using a luminescent up-converting phosphor technology in combination with a lateral flow-based platform (UCP-LF) [23, 24]. A 30 pg/mL cutoff threshold was used. Schistosome infection was defined as having either a positive microscopy or CAA test.

Plasma viral load was quantified using the AmpliPrep/COBAS® TaqMan® HIV-1 Test (Roche Molecular Systems Inc., Pleasanton, California, USA) machine at the BMC clinical laboratory, with a lower limit of detection of 20 copies/mL. Virological failure was defined as a viral load above 1000 copies/mL.

### Statistical analysis

We estimated that we needed to enroll 190 patients to provide 80% power to detect the primary outcome, a predicted prevalence of virological treatment failure of 45% in those with schistosome infection compared to 25% in those without schistosomes, assuming that 30% of the study population would be infected with schistosomes [10]. Data was double entered, verified and cleaned using Microsoft Excel 2013 and analysis was performed using STATA version 13. Chi-square tests and t-tests were used to compare presence of demographic and clinical factors in those with versus without schistosome infection. Univariable and multivariable linear regressions were used to determine factors associated with log_10_ of the viral load, including schistosome infection. All variables significantly associated with the outcome in the univariable analysis were included in the stepwise multivariable analysis. We pre-specified that we would include schistosome status and duration of HIV-1 infection in the model.

We calculated the time delay between HIV infection and start of ART using the normal CD4 decay per calendar year in drug naïve individuals as previously described [19], assuming that the upper reference values of CD4 counts in healthy Tanzanians are 1278.9 cells/μL for men and 1406. 11 cells/μL for women [25]. In addition, we used the overall CD4 decay per calendar year of 34.5 cells/ μL per year that had been reported from Rakai, Uganda, which has a similar HIV-1 clade composition to Mwanza, Tanzania [26–29].

We modeled decay by the square-root function [28], which meant we subtracted 5.87 cells^0.5^/μL^0.5^ from 35.76 cells^0.5^/μL^0.5^ for men and 37.51 cells^0.5^/μL^0.5^ for women per calendar year until the square root of CD4 count found at time of start of ART was reached. The time period for this to happen was considered to be the estimated period between HIV-1 acquisition and start of ART. The time between start of ART and date of viral load testing was then added to this variable to obtain the total duration of HIV-1 infection. This led to an estimated median time from seroconversion to ART initiation of 4.4[3. 11–5.1] years, which is similar to what was found using HIV surveillance data [Colombe et al., manuscript in revision].

### Definitions

Immunological failure was defined as either a confirmed 50% drop from peak CD4 count (if known) during current treatment or as a CD4 count equal to or below baseline after 6-months of ART, or as a CD4 count below 100cells/mm3 after 1 year of ART as per WHO guidelines [30]. Poor ART adherence was defined as missing 3 doses on 2 or more days of ART within the last 3 months according to Tanzanian national guidelines [31]. This measure of adherence is routinely recorded at every monthly visit by nurses and doctors at the clinic [31].

## Results

From August to December 2014, we screened 237 outpatients who were diagnosed with immunological treatment failure while using first-line ART. Of these, 31 were using a stavudine-containing regimen, 10 had been on ART for less than 6 months, 6 did not want to participate in the study, and viral load testing failed on 5 patients. Thus we enrolled and analyzed data from a total of 188 patients.

The median age was 41.0 [36.0–46.5] years and 116 (61.7%) of participants were female. The majority of participants resided in urban areas (152, 80.9%), were employed and selling food (89, 47. 3%), and had completed only primary education (118, 62. 11%). 63/87 (72%) of anemic patients were female (*p* = 0.005). 79/188 (42.0%) of patients had > 1000 viral copies/mL. Among those with viral loads < 1000 copies/mL, 94 (86.2%) were virologically suppressed with viral loads < 20 copies/mL. 85/173 (49.1%) had either a microscopy positive test or a positive CAA test for *Schistosoma* sp. (Table 1). More specifically, 19/170 (11.18%) had a stool microscopy positive test, no one had a urine microscopy positive test and 82/188 (43.62%) had a positive CAA test. Sixteen people were positive both by CAA and microscopy test.

**Table 1:** Demographic characteristics of 188 HIV-1 outpatients with immunological treatment failure on ART

Variable	Viral load > 1000 copies/mL N (%) or Median [IQR] *N* = 79	Viral load < = 1000 copies/mL N (%) or Median [IQR] *N* = 109	*p*-value
Female	53/79 (67.1%)	63/109 (57.8%)	0.20
Age in years	40 [34–46]	42 [37–48]	0.034
Level of education			0.44
Informal	47/79 (59.5%)	71/109 (65.1%)	
Any primary schooling	21/79 (26.6%)	23/109 (21.1%)	
Any secondary schooling	3/79 (3.8%)	1/109 (0.9%)	
Any university	8/79 (10.1%)	14/109 (12.8%)	
Time on ART			0.25
6 m–1y	2/79 (2.5%)	1/109 (0.9%)	
1–2y	6/79 (7.6%)	18/109 (16.5%)	
2–3y	14/79 (17.7%)	16/109 (14.7%)	
>3y	57/79 (72.2%)	74/109 (67.9%)	
ART Regimen			0.77
AZT/3TC/NVP	35/79 (44.3%)	49/109 (45.0%)	
AZT/3TC/EFV	10/79 (12.7%)	19/109 (17.4%)	
TDF/3TC/EFV	22/79 (27.8%)	28/109 (25.7%)	
TDF/3TC/EFV	12/79 (15.2%)	13/109 (11.9%)	
History of change ART Regimen	34/79 (43.0%)	40/109 (36.7%)	0.38
Peak CD4 (cells/μL)	358 [202–506]	450 [317–630]	0.0027
Current CD4 (cells/μL)	104 [47–179]	192 [125–239]	< 0.001
History of stopping ART	19/79 (24.1%)	16/109 (14.7%)	0.10
Use of herbal/alternative medicine	32/79 (40.5%)	54/109 (49.5%)	0.22
Other comorbidities			0.38
None	73/79 (92.4%)	94/109 (86.2%)	
Hypertension	4/79 (5.1%)	8/109 (7.3%)	
Others (Diabetes, heart failure, asthma etc.)	2/79 (2.5%)	7/109 (6.4%)	
Body mass index in kg/m^2^	21.5 [18.9–25.4]	22 [19.9–24.0]	0.65
Anemia (Hemoglobin< 12 g/dl)	50/79 (6.3%)	37/109 (33.9%)	< 0.001
Schistosome positivity	36/71 (50.7%)	49/102 (48.0%)	0.73
Ln of CAA (pg/ml)	3.05 [2.1–5.2]	2.8 [2.0–6.1]	0.65
Years infected with HIV-1	8.9 [6.6–11.1]	9.9 [7.7–11.4]	0.068

After univariable analysis, female sex, lower peak CD4 counts, lower current CD4 counts, anemia, and shorter time infected with HIV-1 were all significantly associated with higher log_10_ of viral load. Schistosome infection was not associated with log_10_ of viral load.

After multivariable analysis, the best fit model included sex and current CD4 counts. We kept duration of HIV-1 infection in the model. Results of both the univariable and multivariable analyses are presented in Table 2. Neither schistosome infection nor duration of HIV-1 infection was associated with log_10_ of viral load. The interaction between sex and schistosome status and its impact on log_10_ of viral load was not statistically significant.

**Table 2:** Results of univariable and multivariable analyses of factors associated with log_10_ of viral load

Variable	Univariable analysis		Multivariable analysis	
	Slope coefficient [95%CI]	*p*-value	Slope coefficient [95%CI]	*p*-value
Female	0.6 [0.07;1.2]	0.029	0.6 [0.05;1.2]	0.034
Age (as a continuous variable)	− 0.02 [− 0.05;0.006]	0.11		
Level of education				
Informal	Ref			
Any primary schooling	0.2 [− 0.5;0.9]	0.56		
Any secondary schooling	−0.08 [− 1.8;1.6]	0.92		
Any university	0.03 [− 0.98;1.04]	0.95		
Time on ART				
6 m-1y	Ref			
1–2y	−1.6 [−4.0;0.8]	0.20		
2–3y	−1.0 [− 3.4;1.4]	0.40		
>3y	− 0.7 [− 3.0;1.6]	0.53		
History of change ART Regimen	0.2 [− 0.4;0.8]	0.42		
Highest/Peak CD4 (cells/μL)				
< 200	Ref			
200–500	−0.8 [− 1.6;-0.05]	0.037		
500–1000	− 1.1 [− 1.9;-0.3]	0.011		
> 1000	−3.0 [− 6.2;0.2]	0.068		
Current CD4 (cells/μL)				
< 100	Ref		Ref	
100–200	− 0.5 [− 1.2;0.08]	0.090	−0.5 [− 1.1;0.2]	0.15
200–300	−1.4 [−2.2;-0.7]	< 0.001	-1.3 [− 2.1;-0.5]	0.001
> 300	-2.8 [−5.0;-0.6]	0.014	-3.0 [− 5.2;-0.8]	0.007
History of stopping ART	−0.05 [− 0.7;0.6]	0.89		
Use of herbal/alternative medicine	−0.1 [− 0.7;0.4]	0.61		
Other comorbidities				
None	Ref			
Hypertension	−0.7 [− 1.9;0.6]	0.30		
Others (Diabetes, heart failure, asthma etc.)	−0.7 [− 2.2;0.8]	0.34		
Anemia	0.8 [0.3–1.4]	0.005		
Schistosome positivity	0.15 [− 0.5;0.8]	0.63	0.1 [−0.4;0.7]	0.68
Ln of CAA (in ln pg/mL)	0.03 [−0.04;0.1]	0.42		
Years infected with HIV-1	0.1 [0.01;0.2]	0.027	0.06 [−0.06;0.2]	0.32

**Fig. 1:**
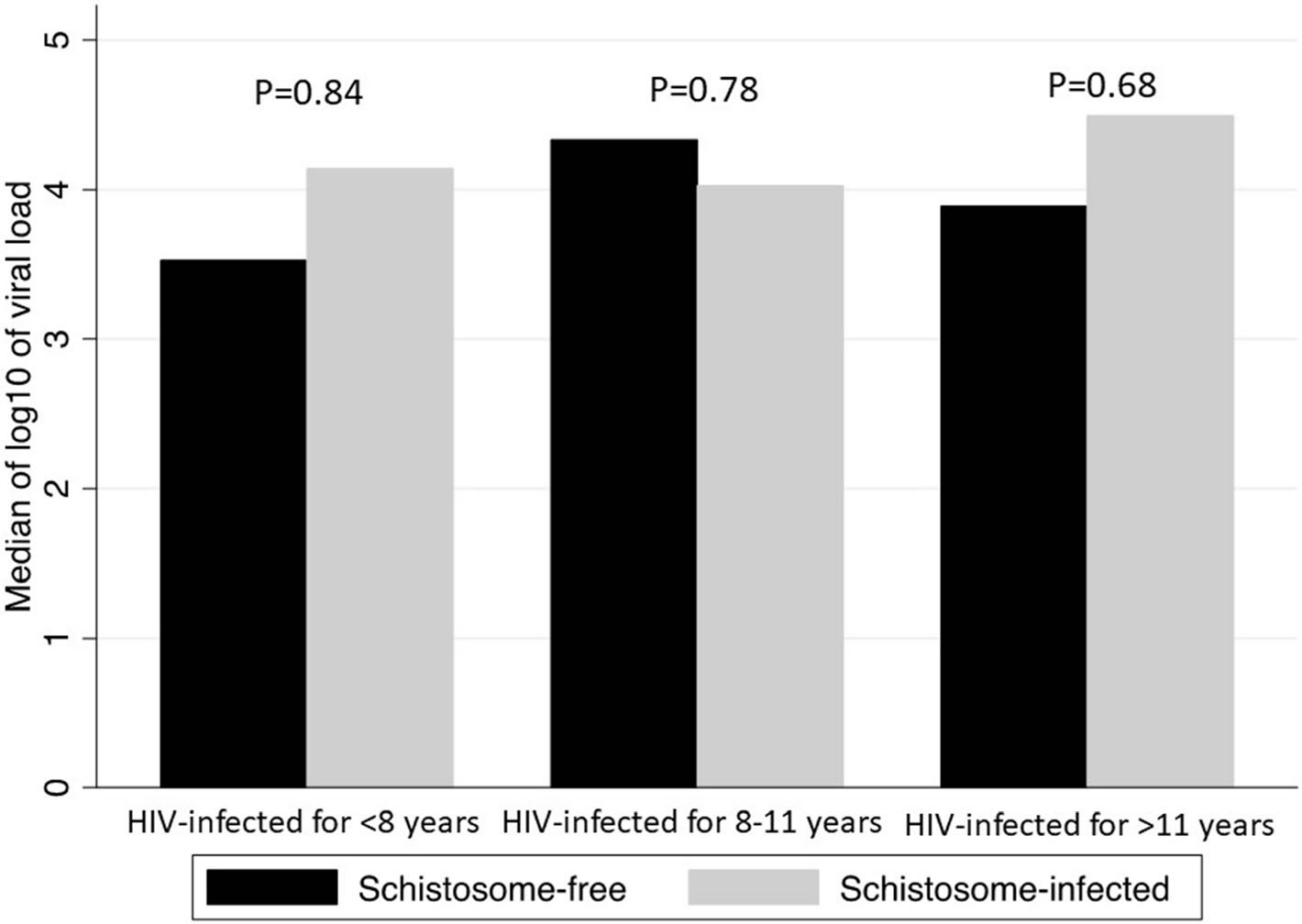
Difference in log_10_ of viral load by schistosome status and time infected with HIV-1. Figure shows no difference in median of log_10_ of viral load between schistosome infected and uninfected patients with immunological failure on ART across time infected with HIV-1

When looking at whether the median log_10_ of viral load between schistosome infected and non-infected patients changed over time, we found no significant differences (Fig. 1).

## Discussion

We found no association between schistosome infection and viral loads among patients with immunological failure, including after adjustment for time infected with HIV-1. This suggests that observed effects of schistosome infection on HIV-1 viral load occur in the absence of ART and that, once ART commences, effective ART is the dominant driver of viral load. In addition, our finding suggests that the increased immunological failure found by Efraim et al. might be due to an effect of *Schistosoma* sp*.* on CD4 counts, but not on viral loads, in people on ART [10].

Our findings, adjusting for duration of HIV-1 infection, differ from those in ART-naïve patients in the same clinic, where schistosome-infection was associated with lower viral loads after adjustment for duration of HIV infection [19]. It is likely that the effect of ART, which typically decreases the viral load by several log_10_ fold copies/mL, is so strong that it would mask any effect of schistosome infection on viral load, which we estimated in our prior study to cause a difference of 0.7 log10 copies/mL in ART-naïve patients. Viral load dynamics of patients on ART appear to be vastly different from those of ART naïve patients, as suggested by the fact that baseline viral loads do not predict viral load rebounds on ART in previously ART-naïve patients [32].

Expectedly, higher peak and current CD4 counts were associated with lower viral load. This finding is consistent with several other studies in sub-Saharan Africa [33, 34]. Similarly the association between anemia and higher viral load has been shown before [35]. The significant association of female sex with higher viral load as observed in our study contradicted what is usually reported [33, 36, 37]. This might be due to the fact that women were significantly more likely to have anemia, which itself is associated with higher viral load and became insignificant when included in the multivariable analysis.

These results need to be considered in light of some limitations. The study included only people with immunological failure, implying that our cohort has overall low CD4 count and thus high viral loads. In addition, as immunological failure is also associated with specific host and viral genetics [38, 39], our overall findings may not directly apply to each individual patient on ART. Finally the sample size was calculated for a statistical analysis based on a binary outcome rather than a continuous one. By using viral load as a continuous variable, we lose some power. Nonetheless, the conclusions when using a binary outcome remain the same (Unadjusted Odds Ratio = 1.11[0.61–2.04], *p* = 0.73; Odds Ratio adjusted for CD4 counts and anemia = 0.99[0.50–1.95], *p* = 0.98).

## Conclusions

In conclusion, our study suggests that schistosome infection does not impact viral load in HIV-1 infected patients immunologically failing on ART, likely due to the major effects of ART on viral load once ART is initiated.

## Abbreviations

95%CI: 95% confidence interval
ART: Antiretrovial therapy
BMC: Bugando Medical Centre
CAA: Schistosome circulating anodic antigen
CD4: CD4+ T-cells
IQR: Interquartile range
N: Total number of observations
Th: T-helper
UCP-LF: Luminescent up-converting phosphor technology in combination with a lateral flow-based platform
WHO: World Health Organization
